# Exploiting the damaging effects of ROS for therapeutic use by deactivating cell-free chromatin: the alchemy of resveratrol and copper

**DOI:** 10.3389/fphar.2024.1345786

**Published:** 2024-02-22

**Authors:** Indraneel Mittra

**Affiliations:** ^1^ Translational Research Laboratory, Advanced Centre for Treatment, Research and Education in Cancer, Tata Memorial Centre, Navi Mumbai, India; ^2^ Homi Bhabha National Institute, Navi Mumbai, India

**Keywords:** cell-free chromatin particles, chemotherapy toxicity, copper, reactive oxygen species, resveratrol, sepsis

## Abstract

Cell-free chromatin particles (cfChPs) that circulate in blood, or those that are released locally from dying cells, have myriad pathological effects. They can horizontally transfer themselves into healthy cells to induce DNA damage and activate inflammatory and apoptotic pathways. It has been proposed that repeated and lifelong assault on healthy cells by cfChPs may be the underlying cause of ageing and multiple age related disorders including cancer. The damaging effects of cfChPs can be minimized by deactivating them via the medium of ROS generated by admixing the nutraceuticals resveratrol (R) and copper (Cu). The antioxidant R acts as a pro-oxidant in the presence of Cu by its ability to catalyse the reduction of Cu(II) to Cu(I) with the generation of ROS via a Fenton-like reaction which can deactivate extra-cellular cfChPs. This perspective article explores the possibility of using the damaging potential of ROS for therapeutic purposes. It discusses the ability of ROS generating nutraceuticals R-Cu to deactivate the extracellular cfChPs without damaging effects on the genomic DNA. As cfChPs play a key role in activation of various disease associated pathways, R-Cu mediated deactivation of these pathways may open up multiple novel avenues for therapy. These findings have considerable translational implications which deserve further investigation by the way of well-designed randomised clinical trials.

## 1 Introduction

Reactive oxygen species (ROS) generated due to the incomplete reduction of molecular oxygen is known to exert various damaging effects on DNA, proteins, and lipids (Checa and Aran, 2020). However, two unrelated discoveries have indicated that ROS can be repurposed for therapeutic use. The first discovery revealed that cell-free chromatin particles (cfChPs) released from the billions of dying cells can readily enter into the healthy cells. These cfChPs can induce DNA double strand (dsDNA) breaks and activate inflammatory as well as apoptotic pathways (Mittra et al., 2015). It was hypothesised that lifelong interaction of healthy cells with cfChPs is the underlying cause of multiple disease conditions, including ageing and cancer (Raghuram et al., 2019). The second discovery showed that ROS can be generated *in vivo* by admixing the nutraceuticals resveratrol and copper (R-Cu), which can potentially mitigate the various pathological effects of cfChPs (Fukuhara et al., 2006). Overall, these two discoveries collectively suggest the probable use of the nutraceuticals resveratrol and copper (R-Cu), for therapeutic use.

In general, ROS are highly reactive unstable form of oxygen moieties containing one or more unpaired electrons in their outer shells (Jomova et al., 2023). Under normal physiological conditions and redox homeostasis, these molecules act as vital signalling mediators. The levels of ROS are continuously monitored by the cell and maintained at physiological levels by a complex antioxidant defence system which is designed to protect the cell from the toxicity associated with excessive ROS. This includes enzymatic systems such as superoxide dismutase, glutathione peroxidase, and catalase (Finkel and Holbrook, 2000). The imbalance between ROS production and antioxidant defences results in generation of oxidative stress, which has been recognized as a key inducer of various process resulting in aging, cancer and other number of chronic diseases (Valko et al., 2007; Liguori et al., 2018).

cfChPs that are released from dying cells have been shown to circulate in the blood of healthy individuals and, at higher levels, in individuals with a range of acute and chronic disease states, such as cancer (for a review, see Mittra et al., 2012). Data emerging from previous studies suggest that cfChPs have wide-ranging local and systemic pathophysiological effects. cfChPs can readily enter into healthy cells, localise within their nuclei and associate themselves with the host cell chromosomes to integrate and damage their DNA. These steps also result in activation of inflammatory and apoptotic pathways (Mittra et al., 2015; 2017b).

The above findings highlight the need to exploit the damaging effects of ROS for therapeutic use by deactivating cell-free chromatin potentially leading to amelioration of multiple pathological conditions.

## 2 *In vivo* generation of ROS for therapeutic use

**FIGURE 1:**
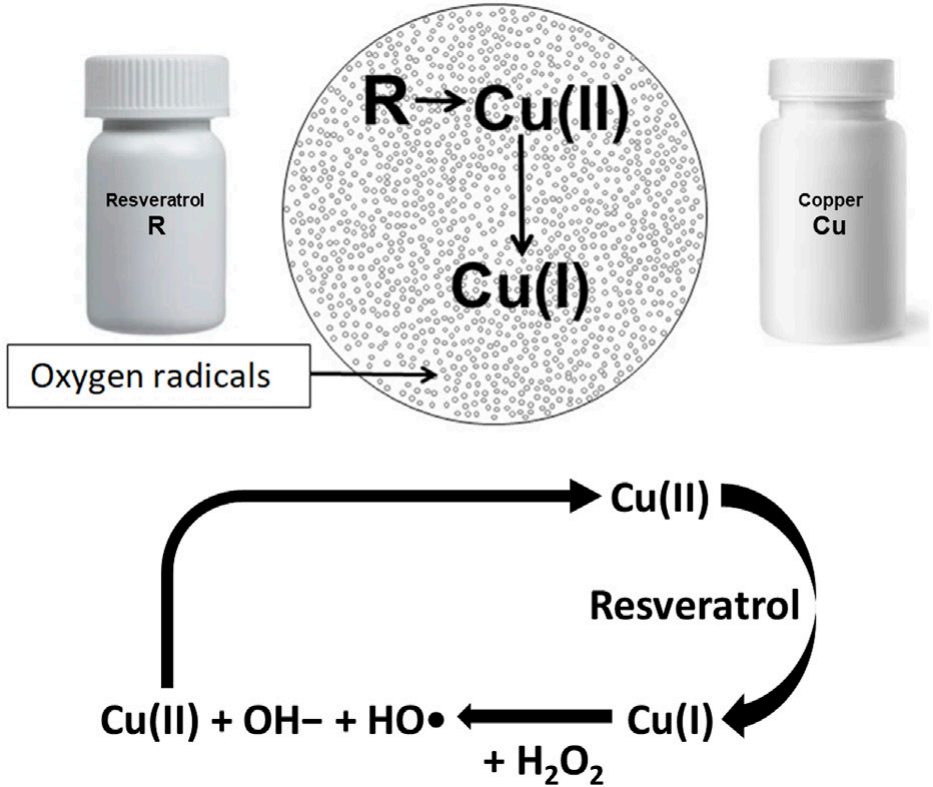
Admixing resveratrol and copper generates oxygen radicals via a Fenton like reaction. Resveratrol catalyses the reduction of Cu(II) to Cu(I); the latter reacts with hydrogen peroxide (H_2_O_2_), leading to the formation of hydroxide ions (OH−), and a hydroxyl radical (OH) and also regenerates Cu(II) in a cyclic manner.

The seminal paper by Fukuhara and Miyata, (1998) showed that oxygen radicals are generated when R and Cu are admixed (Figure 1). They reported that R reduces Cu (II) to Cu (I) resulting in generation of oxygen radicals via a Fenton-like reaction (Li et al., 2018). Fukuhara’s group also showed that oxygen radical thus generated can cleave plasmid DNA (Fukuhara et al., 2006). Further research has shown that cfChPs can be deactivated by the ROS generated by admixing small quantities of the nutraceuticals resveratrol (R) and copper (Cu) (Kirolikar et al., 2018). Resveratrol is a plant polyphenol that is recognized for its antioxidant and radical-scavenging activities (Gülçin, 2010). When administered orally, R catalyses the reduction of Cu(II) to Cu(I), which is associated with ROS generation in the stomach ostensibly by a Fenton-like reaction (Li et al., 2018). The ROS are readily absorbed and lead to systemic effects in the form of deactivation of extracellular cfChPs. It has been further reported that reduction in molar concentration of Cu with respect to R increases the cfChP-degrading activity of R–Cu (Subramaniam et al., 2015).

## 3 R-Cu treatment retards ageing and neurodegeneration

**FIGURE 2:**
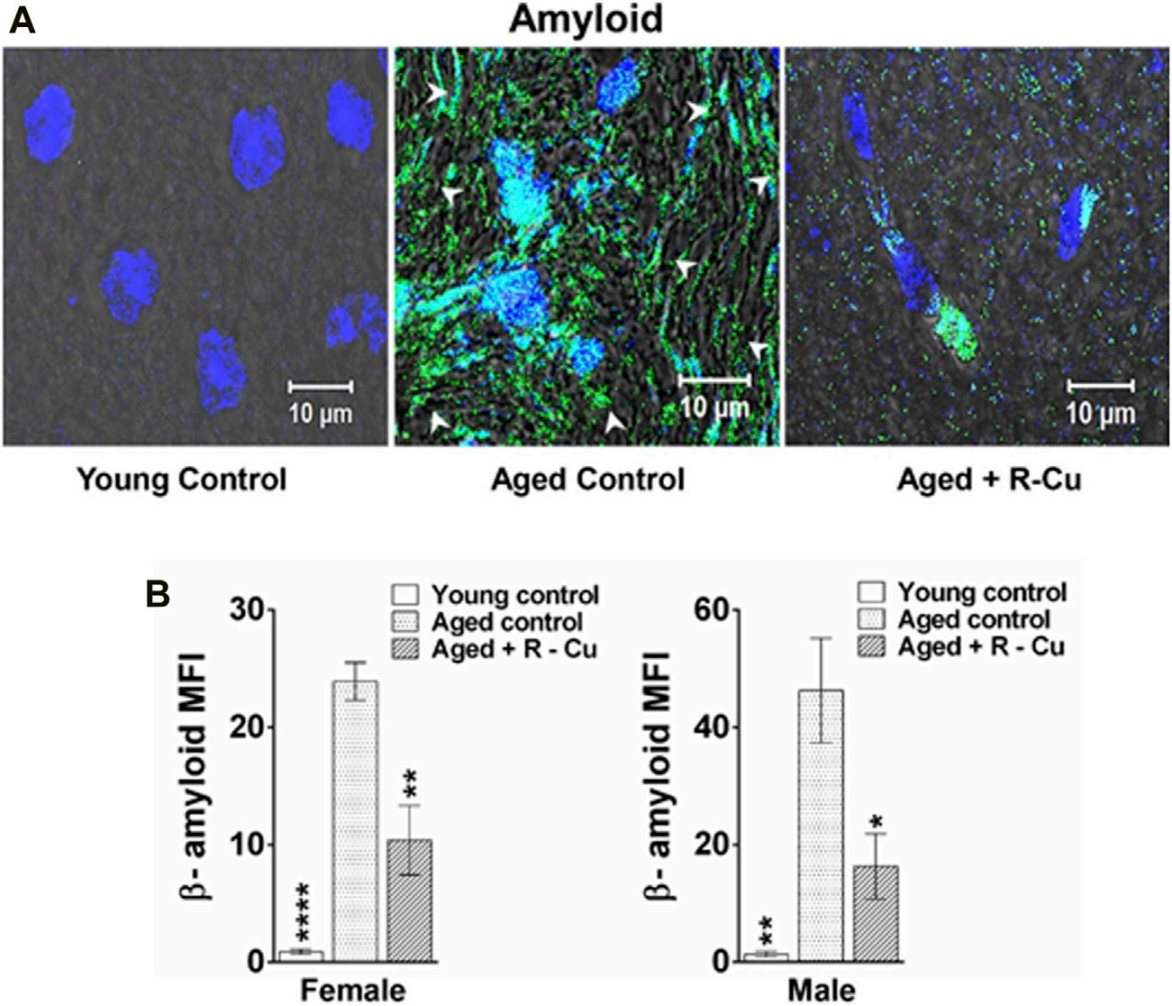
Prolonged oral treatment with R-Cu markedly reduces amyloid deposition in ageing mouse brain cells. Twenty-four C57Bl/6 mice were divided into 3 equal groups. Mice in the first group were killed at age 3 months and served as young controls. Those in the other two groups were allowed to age and, at age 10 months, the experimental group was given R-Cu (R = 1 mg/kg and Cu = 0.1 μg/kg) twice daily by oral gavage for a further 12 months. Mice in the control ageing group were given water twice daily by oral gavage for 12 months. Animals of both groups were sacrificed at age 22 months. **(A)** Representative confocal images of a mouse brain showing reduced amyloid deposition in an aged mouse given R-Cu **(B)** Quantitative representation of amyloid deposition in the three groups of mice. *p < 0.05; **p < 0.01; ****p < 0.0001 as analysed by two-tailed Student’s t-test (Reproduced from Pal et al. (2022) under Creative Commons License).

The results of available animal and human studies on the use of antioxidant therapies to slow ageing have been conflicting and disappointing (Iakovou and Kourti, 2022). However, compelling recent evidence indicates that ageing is the result of the lifetime damaging effects of cfChPs on healthy cells which can be ameliorated by ROS generated by the administration of R-Cu. It has been recently reported that the prolonged oral administration of R-Cu can downregulate multiple biological hallmarks of ageing in brain cells of C57Bl/6 mice, including telomere shortening, amyloid deposition, apoptosis, inflammation, mitochondrial dysfunction, DNA damage, senescence, and aneuploidy (Figure 2; Pal et al., 2022). In the same study, R-Cu administration was also associated with significant reductions in the levels of serum glucose, cholesterol, and C-reactive protein (Pal et al., 2022). An interesting observation was the differential effects of R-Cu between male and female mice on telomere shortening, which requires further study (Pal et al., 2022). It is possible that the reported anti-ageing effects of R alone are contingent on the presence of small quantities of Cu, and that the conflicting results reflect the inconsistent availability of Cu in the stomach of the study subjects. Lack of Cu availability would impair R’s sustained ROS-generating activity and hamper the deactivation of extracellular cfChPs that is required for the anti-ageing effects of R-Cu to be realized.

## 4 R-Cu treatment minimizes chemotherapy toxicity

### 4.1 Pre-clinical studies

The benefits of R-Cu have also been demonstrated with respect to cancer chemotherapy. It is a commonly held belief that the toxicity of chemotherapy is a direct damaging effect of the drugs on healthy cells, despite the manifestations of such toxicity far outlasting the half-life of most chemotherapeutic agents, which is approximately 24 h (Chu and DeVita, 2012). For example, bone marrow suppression following chemotherapy reaches a nadir after 7–14 days, but the neutropenia that follows often lasts for more than 3 weeks. Challenging the dogma that chemo-toxicity is a direct result of drug assault, it has been shown that chemotherapy induced toxic side-effects are primarily caused by cfChPs that emerge from the first round of therapy-induced dying cells. This set in motion a vicious cycle of further cell death and inflammation that exaggerates and prolong the toxic effects. The latter can be prevented by the concurrent administration of R-Cu with chemotherapy, as evidenced by marked downregulation of the dsDNA damage marker γ-H2AX and the inflammatory cytokines NFκB, IL-6, TNF-α, and INF-γ in all organs and tissues examined (Mittra et al., 2017a).

### 4.2 Clinical studies

The above pre-clinical studies have been replicated in two clinical trials of high dose chemotherapy in patients receiving bone marrow transplantation (Agarwal et al., 2022) and in patients with advanced gastric cancer (Ostwal et al., 2022). In the first study (Agarwal et al., 2022), 25 patients receiving haematopoietic stem cell transplantation for multiple myeloma were administered two different doses of R-Cu (R = 5.6 mg and Cu = 560 ng, or R = 50 mg and Cu = 5 μg). R-Cu was administered orally twice daily, starting 48 h before the administration of melphalan chemotherapy and continued until 21 days post-transplantation. An assessment of grade 3 or higher toxicity with respect to the commonly reported adverse effects of high dose chemotherapy, namely, mucositis, nausea, vomiting, diarrhoea, opioids dependence, and the need for total parenteral nutrition, was performed. The incidence of grade 3 mucositis was found to be significantly reduced; also reduced were the levels of TNF-α and IL-1β. However, the reduction in the incidence of other toxic effects was not statistically significant (Agarwal et al., 2022).

The second study was a prospective, phase II, single-arm open-label study conducted to investigate the reduction in haematological and non-haematological toxicities in 30 patients with advanced gastric cancer during docetaxel-based multi-agent chemotherapy (Ostwal et al., 2022). Patients were orally administered R-Cu (R = 5.6 mg and Cu = 560 ng) three times daily concurrently with chemotherapy for 6 months or until progression of disease, whichever occurred earlier. The incidence of serious and troublesome non-haematological toxicities, such as hand-foot syndrome, diarrhoea, and vomiting, was dramatically reduced in the R-Cu group compared to that in historical controls. However, although the cumulative incidence of overall and haematological toxicities also declined, they failed to attain statistical significance (Ostwal et al., 2022).

## 5 R-Cu treatment reverses aggressive biological features of cancer

Another study investigated whether ROS generated following the oral administration of R-Cu would lead to biological effects on cancer cells in terms of downregulation of the hallmarks of cancer and immune checkpoints. The study was conducted in 25 patients with advanced squamous cell carcinoma of the oral cavity (Pilankar et al., 2022). It was observed that cfChPs from dying cancer cells that had entered the tumour microenvironment could markedly activate the hallmarks of cancer and immune check-points in surviving cancer cells (Pilankar et al., 2022). Oral treatment with R-Cu was associated with the significant downregulation of 21 out of 23 biomarkers, representing the 10 hallmarks of cancer and immune checkpoints defined by Hanahan and Weinberg (2011). These results suggested that R-Cu-generated ROS can reverse the aggressive biological features of cancer raising the formidable possibility that prolonged R-Cu treatment could lead to the healing of cancer.

## 6 R-Cu treatment prevents potential metastatic spread of cancer

In a recent article it was shown that all three forms of traditional therapeutic interventions for cancer, namely, chemotherapy, radiotherapy, and surgery promote the systemic dissemination of cancer via release of cfChPs from therapy-induced dying cancer cells (Raghuram et al., 2023). The treatment of human breast cancer xenografts in severely immunodeficient mice with chemotherapy, localised radiotherapy, or surgical excision was shown to lead to the systemic dissemination of human oncogenes detectable in mouse brain cells (Raghuram et al., 2023). This raises the possibility that contemporary cancer treatments can potentially facilitate spread of the disease, a concern that has been raised in a number of previous publications (Demicheli et al., 2008; Vilalta et al., 2016; Karagiannis et al., 2017; Martin et al., 2017; Tohme et al., 2017; Monteran et al., 2022). However, the simultaneous administration of the cfChPs deactivating agent R-Cu with all three forms of therapeutic interventions for cancer dramatically reduced the systemic spread of oncogenes, ostensibly via the medium of ROS (Raghuram et al., 2023).

## 7 R-Cu treatment downregulates immune checkpoints *in vitro*

Immune checkpoints are protein molecules expressed on immune cells which prevent the immune system from attacking self-cells. Cancer cells being immunologically altered, can protect themselves from attack by activating immune (Zhang and Zheng, 2020). Targeting activated immune checkpoints with specific inhibitors is, therefore, being widely used in the treatment of cancer (Jiang et al., 2021). In spite of much interest in immune checkpoint biology and cancer therapy, how immune checkpoints are activated has not been elucidated. It has been recently reported that cfChPs released from dying cells are the activators of the immune check-points PD-1, CTLA-4, LAG-3, NKG2A, and TIM-3 in human lymphocytes (Shabrish et al., 2024). Notably, it was found that activation of these immune check-points could be abrogated *in vitro* through the administration of R-Cu (Shabrish et al., 2024).

## 8 R-Cu treatment prevents endotoxin-induced sepsis

**FIGURE 3:**
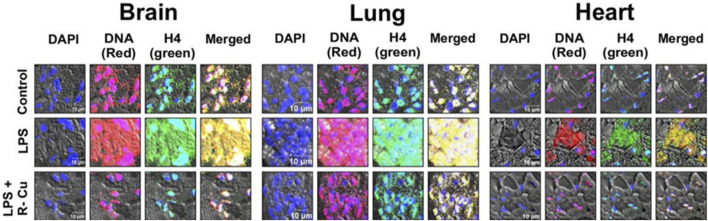
Representative confocal images of mice brain, lung, and heart showing eradication of extracellular cfChPs in LPS-treated mice concurrently given R-Cu. Sections of mice brain, lung, and heart were fluorescently immunostained with fluorescent antibodies against DNA and histone H4 prior to examination via confocal microscopy. Co-localizing DNA (red) and histone H4 (green) fluorescent signals generate yellow / white particles representing cfChPs. (Reproduced from Mittra et al. (2020b) under Creative Commons License).

Evidence of the benefits of R-Cu on the effects of cfChPs is not limited to ageing and cancer. Sepsis is defined as life-threatening organ dysfunction caused by a dysregulated host response to infection (Singer et al., 2016). Despite extensive efforts by the scientific community, the pathophysiology of sepsis remains poorly understood. However, a study of endotoxin sepsis in mice demonstrated that sepsis follows a similar cascading vicious cycle of cfChP-induced cell death and inflammation following microbial infection (Mittra et al., 2020b). Specifically, the administration of R-Cu alongside lipopolysaccharide (LPS)—an agent commonly used to induce experimental sepsis in mice—was found to prevent a number of pathological events, including the release of cfChPs in the extracellular spaces of the brain, lung, and heart. In addition, treatment with R-Cu restricted the onset of multiple adverse pathways such as DNA damage, apoptosis, and inflammation in multiple organs and tissues. It also prevented kidney dysfunction, elevation of serum lactate levels; and coagulopathy, fibrinolysis, as well as thrombocytopenia (Figure 3; Mittra et al., 2020b). Finally, R-Cu-generated ROS could prevent LPS-induced fatality in mice. These promising findings deserve replication in clinical studies. Indeed, in an observational study in patients with severe coronavirus disease 2019, it was found that R-Cu treatment reduced mortality by nearly 50% when compared to historical controls (Mittra et al., 2020a).

## 9 Absence of harmful side effects of R-Cu treatment

No harmful side effects in the form of damage to genomic DNA were reported in any of the above pre-clinical and clinical studies. ROS generated following oral administration of R-Cu led to markedly upregulated antioxidant defence systems which ostensibly neutralised the invading oxygen radicals and prevented damage to cellular genomic DNA (Pal et al., 2022; Pilankar et al., 2022). This was substantiated by the finding of reduced γ-H2AX signals in brain cells following prolonged R-Cu administration to aged mice (Pal et al., 2022). This implied that the body’s upregulated antioxidant enzymes shielded every cell’s genome from the potentially harmful effects of ROS.

## 10 Future directions

cfChPs, which are released from apoptotic cells have been shown to exist in circulation and at higher levels, in diseased individuals. As cfChPs can have wide-ranging pathophysiological effects, designing novel strategies that can reduce the load of cfChPs can be an important treatment option for multiple diseases. Moreover, although ROS act as a mediator of various critical signalling pathways. excessive ROS production can exert a number of damaging effects on DNA, proteins and lipids (Liguori et al., 2018). Studies have also recognized ROS associated oxidative stress as a critical factor in the pathogenesis of multiple chronic diseases such as Alzheimer’s disease, Parkinson’s disease, diabetes as well as aging and cancer (Valko et al., 2007; Manoharan et al., 2016). Significantly, these damaging effects of ROS can be exploited for therapeutic purposes. The present article shows that the ROS generating nutraceuticals R and Cu can be used to reduce the load of cfChPs and can have multiple therapeutic effects. These observations suggest that R-Cu possess the ability to deactivate extracellular cfChPs in a variety of disease conditions without damaging effects on the genomic DNA. The findings discussed in this article have considerable clinical and translational implications which merit further investigations in the form of well-designed randomised clinical trials.

## Data Availability

The original contributions presented in the study are included in the article/Supplementary material, further inquiries can be directed to the corresponding author.
